# Validation of semaphorin 7A and ala-β-his-dipeptidase as biomarkers associated with the conversion from clinically isolated syndrome to multiple sclerosis

**DOI:** 10.1186/s12974-014-0181-8

**Published:** 2014-11-13

**Authors:** Ester Cantó, Mar Tintoré, Luisa Maria Villar, Eva Borrás, Jose Carlos Álvarez-Cermeño, Cristina Chiva, Eduard Sabidó, Alex Rovira, Xavier Montalban, Manuel Comabella

**Affiliations:** Servei de Neurologia-Neuroimmunologia, Centre d’Esclerosi Múltiple de Catalunya (Cemcat), Institut de Recerca Vall d’Hebron (VHIR), Hospital Universitari Vall d’Hebron, Universitat Autònoma de Barcelona, Ps. Vall d’Hebron 119-129, 08035 Barcelona, Spain; Department of Neurology and Immunology, Hospital Ramón y Cajal, Ctra. de Colmenar Viejo, km. 9,100, 28034 Madrid, Spain; Proteomics Unit, Centre for Genomic Regulation (CRG) and Universitat Pompeu Fabra (UPF), C. Doctor Aiguader 88, 08003 Barcelona, Spain; Unitat de RM, Servei de Radiologia, Hospital Universitari Vall d’Hebron, Universitat Autònoma de Barcelona, Ps. Vall d’Hebron 119-129, 08035 Barcelona, Spain; Unitat de Neuroimmunologia Clínica, Cemcat, Hospital Universitari Vall d’Hebron, Pg. Vall d’Hebron 119-129, 08035 Barcelona, Spain

**Keywords:** Multiple sclerosis, Clinically isolated syndrome, Biomarkers, Cerebrospinal fluid, Conversion to MS

## Abstract

**Background:**

In a previous proteomics study using pooled cerebrospinal fluid (CSF) samples, we proposed apolipoprotein AI, apolipoprotein AIV, vitronectin, plasminogen, semaphorin 7A, and ala-β-his-dipeptidase as candidate biomarkers associated with the conversion to clinically definite multiple sclerosis (CDMS) in patients with clinically isolated syndromes (CIS). Here, we aimed to validate these results in individual CSF samples using alternative techniques.

**Methods:**

In a first replication study, levels of apolipoproteins AI and AIV, vitronectin, and plasminogen were measured by ELISA in CSF and serum of 56 CIS patients (29 patients who converted to CDMS (MS converters) and 27 patients who remained with CIS during follow-up (MS non-converters)) and 26 controls with other neurological disorders. Semaphorin 7A and ala-β-his-dipeptidase levels were determined by selected reaction monitoring (SRM) in CSF of 36 patients (18 MS converters, 18 non-converters) and 20 controls. In a second replication study, apolipoprotein AI levels were measured by ELISA in CSF of 74 CIS patients (47 MS converters, 27 non-converters) and 50 individual controls, and levels of semaphorin 7A and ala-beta-his-dipeptidase were determined by SRM in 49 patients (24 MS converters, 25 non-converters) and 22 controls.

**Results:**

CSF levels of apolipoprotein AI were increased (*P* = 0.043) and levels of semaphorin 7A and ala-β-his-dipeptidase decreased (*P* = 4.4 × 10^−10^ and *P* = 0.033 respectively) in MS converters compared to non-converters. No significant differences were found in serum levels for apolipoproteins AI and AIV, vitronectin, and plasminogen. Findings with semaphorin 7A and ala-β-his-dipeptidase were also validated in the second replication study, and CSF levels for these two proteins were again decreased in MS converters versus non-converters (*P* = 1.2 × 10^−4^ for semaphorin 7A; *P* = 3.7 × 10^−8^ for ala-β-his-dipeptidase). Conversely, apolipoprotein AI findings were not replicated and CSF levels for this protein did not significantly differ between groups. Furthermore, CSF semaphorin 7A levels were negatively associated with the number of T2 lesions at baseline and one-year follow-up.

**Conclusions:**

These results validate previous findings for semaphorin 7A and ala-β-his-dipeptidase, and suggest that these proteins play a role as CSF biomarkers associated with the conversion to CDMS in CIS patients.

**Electronic supplementary material:**

The online version of this article (doi:10.1186/s12974-014-0181-8) contains supplementary material, which is available to authorized users.

## Background

In patients with clinically isolated syndromes (CIS), magnetic resonance imaging (MRI) abnormalities and the presence of immunoglobulin G (IgG) oligoclonal bands in cerebrospinal fluid (CSF) are important predictors of later conversion to multiple sclerosis (MS) [1-4]. With the aim to identify additional molecular biomarkers associated with the conversion to MS, we recently conducted a mass spectrometry-based proteomic study in pooled CSF samples from patients with CIS who converted to clinically definite MS (CDMS) and patients who remained with CIS [5]. Three candidates, chitinase 3-like 1 (CHI3L1), ceruloplasmin, and vitamin D-binding protein were selected for validation in individual CSF samples, and CHI3L1 findings were confirmed in additional cohorts of CIS patients [5]. In this initial screening proteomic study, other proteins were identified and proposed to be candidate biomarkers associated with conversion to MS: apolipoprotein AI (apoAI), apolipoprotein AIV (apoAIV), vitronectin, and plasminogen were found to be upregulated, and semaphorin 7A (sema7A) and ala-beta-his-dipeptidase (CNDP1) were found to be downregulated in CSF pools of CIS patients who converted to CDMS [5]. In the present study, we pursued the validation of these proteins as biomarkers using different techniques in individual CSF samples from CIS patients classified according to their conversion (or lack of) to CDMS.

## Materials and methods

### Patients

Individuals included in the study were part of a cohort of patients with CIS recruited at the Centre d’Esclerosi Múltiple de Catalunya (Cemcat, Barcelona, Spain) from 1995 onwards. The study was approved by the local ethics committee (PR(AG)28/2007). Clinical, CSF, and MRI evaluations have been previously described elsewhere [2]. Patients with CIS were classified according to the following criteria: no conversion to CDMS during the follow-up period, negative IgG oligoclonal bands, and 0 Barkhof criteria at a baseline brain MRI (CIS → CIS group); or conversion to CDMS, presence of IgG oligoclonal bands, and an abnormal brain MRI at baseline (2, 3, or 4 Barkhof criteria) (CIS → CDMS group). A summary of clinical information and CSF characteristics of CIS patients included in the study is shown in Table 1.

**Table 1:** **Clinical information and CSF characteristics of CIS patients included in the study**

	Cohort 1* - ELISA studies			Cohort 2** - proteomic studies		
**Characteristics**	**CIS → CIS**	**CIS → CDMS**	***P*** **values**	**CIS → CIS**	**CIS → CDMS**	***P*** **values**
n	27	29	-	18	18	-
Age (years)^a^	28.1 (9.6)	27.4 (6.3)	0.724	31.7 (7.3)	30.2 (5.9)	0.308
Female/male (% female)	20/7 (74.1)	20/9 (69.0)	0.672	13/5 (72.2)	13/5 (72.2)	1
Follow-up time (years)^a^	8.2 (4.2)	8.6 (3.3)	0.819	6.1 (3.6)	8.8 (2.7)	0.098
Clinical presentation						
Optic neuritis	15 (55.6)	8 (27.6)	0.206	9 (50.0)	3 (16.7)	0.152
Brainstem	4 (14.8)	7 (24.1)		2 (11.1)	5 (27.8)	
Spinal	5 (18.5)	8 (27.6)		3 (16.7)	6 (33.3)	
Others	3 (11.1)	6 (20.7)		4 (22.2)	4 (22.2)	
CSF cells^b^	0.5 (0-5.0)	2.0 (0-9.0)	0.337	0 (2.4-3.5)	4.0 (0-11.5)	0.059
Proteins (mg/dL)^b^	33.0 (24.0-43.0)	37.0 (26.8-57.3)	0.391	32.5 (23.7-41.5)	29.5 (24.0-51.8)	0.705

*Cohort 1 was used for the determination of CSF and serum levels of apolipoprotein AI, apolipoprotein AIV, vitronectin, and plasminogen by ELISA. **Cohort 2 was used for the quantification of CSF levels of semaphorin 7A and ala-beta-his-dipeptidase by selected reaction monitoring (SRM). A total of 23 (63.9%) patients were present in both cohorts of patients. ^a^Data are expressed as mean (standard deviation). ^b^Data are expressed as median (interquartile range). *P* values were obtained following comparisons between CIS → CIS patients and CIS → CDMS patients by means of the chi-square test (gender and clinical presentation) and the Mann-Whitney U test (remaining variables). Proteins (mg/dL) refers to protein concentration in mg/dL. CDMS, clinically definite multiple sclerosis; CIS, clinically isolated syndromes, CSF, cerebrospinal fluid.

Since 2001, a baseline brain MRI scan was performed at the time of the CIS and at 3 to 5 months from disease onset. Follow-up MRI scans were performed at 12 months and every five years after the CIS. The scans were obtained on a 1.5 Tesla (T) magnet until 2009 and on a 3.0 T with a standard head coil since 2010. The following sequences of the brain were performed in each patient: transverse proton density/T2-weighted fast spin-echo, transverse T2-weighted fast-fluid-attenuated-inversion recovery, and transverse T1-weighted spin-echo (600/12/2 (TR/TE/acquisitions)). The transverse T1-weighted sequence was repeated in those patients with demonstrated focal white matter lesions on T2-weighted sequences after gadolinium (Gd) injection (0.1 mmol/kg; scan delay, 5 minutes). The number and location of T2 lesions, number of gadolinium-enhancing lesions, and number of new T2 lesions on the brain were scored. For the number of T2 lesions, three different categories were considered: 0, 1 to 9, and 10 or more lesions.

### Quantification of cerebrospinal fluid and serum levels of apolipoprotein AI, apolipoprotein AIV, vitronectin, and plasminogen

CSF and serum levels of apoAI, apoAIV, vitronectin, and plasminogen were determined in a cohort of 56 CIS patients, 29 of whom converted to CDMS and 27 who remained with CIS (cohort 1; Table 1). A control group of 26 patients with other neurological disorders (OND) was also included in the study (mean age (standard deviation) = 37.8 (14.2) years; 57.1% females; cohort 1, Additional file 1: Table S1). Twenty (35.7%) CIS patients from cohort 1 were also used in the original pooled cohort [5]. Protein levels were determined by commercially available ELISA assays. Levels of apoAI were measured with the ELISAPRO kit for human apolipoprotein AI (3710-1HP-2; Mabtech AB, Nacka Strand, Sweden) following 1:100 and 1:100000 dilutions in CSF and serum samples respectively. Levels of apoAIV were measured with the Human Apolipoprotein AIV ELISA kit (EZHAP0A4-73 K; Millipore Corporation, Billerica, Massachusetts, United States) in undiluted CSF samples and following a 1:500 dilution in serum samples. Levels of vitronectin were quantified with the Human Vitronectin Total Antigen Assay (HVNKT-TOT; Dunn Labortechnik GmbH, Asbach Germany) following 1:50 and 1:50000 dilutions in CSF and serum samples respectively. Levels of plasminogen were measured with the AssayMax Human Plasminogen ELISA Kit (EP1200-1; Assaypro, St Charles, Massachusetts, United States) following 1:100 and 1:20000 dilutions in CSF and serum samples respectively. All samples were measured in duplicate. Respective intra-assay and inter-assay variabilities were 5.3% and 15.0% for apoAI, 6.1% and 21.2% for apoAIV, 2.8% and 15.2% for plasminogen, and 5.1% and 14.1% for vitronectin.

### Quantification of cerebrospinal fluid levels of semaphorin 7A and ala-beta-his-dipeptidase

CSF levels of sema7A and CNDP1 were determined by SRM in a cohort of 36 CIS patients (18 who converted to CDMS and 18 who remained as CIS (cohort 2; Table 1)), and in 20 patients with OND (mean age = 41.4 (15.4); 45% females; cohort 2, Additional file 1: Table S1). Ten (27.8%) CIS patients from cohort 2 also participated in the original pooled cohort [5]. CSF samples were precipitated in acetone overnight at 4°C, solubilized in 6 M urea (Sigma-Aldrich, St. Louis, MO, USA) in 200 mM ammonium bicarbonate (Sigma-Aldrich, St. Louis, MO, USA), reduced with 100 mM dithiothreitol (Sigma-Aldrich, St. Louis, MO, USA), alkylated with 200 mM iodoacetamide (Sigma-Aldrich, St. Louis, MO, USA), digested with endopeptidase Lys-C (Wako Chemicals, Richmond, VA, USA) (2 M urea in 200 mM ammonium bicarbonate at 37°C for 16 hours) and trypsin (Promega, Madison, WI, USA) (1 M urea in 200 mM ammonium bicarbonate at 37°C for 16 hours). After digestion samples were acidified with 10% formic acid and desalted in C18 columns (macro-spin columns, The Nest Group Inc., Southborough, Massachusetts, United States). Four reference isotopically labeled peptides at C-terminal lysine (^13^C_6_,^15^ N_2_-Lys) or arginine (^13^C_6_,^15^ N_4_-Arg) (they are added as a reference since they allow the unequivocal identification of the peptide for the protein of interest) were spiked into the digested samples, two corresponding to sema7A (IFAVWK; VYLFDFPEGK) and two to CNDP1 (ALEQDLPVNIK; HLEDVFSK). Peptides were separated chromatographically with a nanoLC Eksigen coupled to a Q-Trap mass spectrometer (5500 Q-Trap ABSCIEX, Framingham, Massachusetts, United States). Briefly, peptides were initially trapped in a pre-column Acclaim PepMap 100 (C18, 15 μm, 100 Å, Acclaim PepMap 100 ThermoFisher Scientific (Waltham, Massachusetts, United States)) and then separated by reverse-phase chromatography using a 15 cm C18 column (75 μm, Nikkyo Technos Co., Tokyo, Japan) with a gradient of 2 to 40% of solvent B in 35 minutes at a flow rate of 300 nL/min*.* Solvent A: H_2_O, 0.1% formic acid; Solvent B: Acetonitrile, 0.1% formic acid.

SRM acquisition was performed using an unscheduled targeted acquisition method with a dwell time of 20 ms and a total cycle time of 1.4 seconds. For each peptide, 2 to 4 transitions were monitored for both the endogenous (light) and the reference (heavy) forms (Additional file 2: Table S2). SRM data was processed using the Skyline software v1.4.0 (MacCoss lab open-source software, Seattle, United States) [6] and data peaks were evaluated based on retention time, transition intensity rank, and co-elution of the endogenous and reference peptide.

### Validation cohorts

Two additional and totally independent validation cohorts of CIS patients were used to replicate findings with CSF apoAI, sema7A and CNDP1. These CIS patients were classified into non-converters and converters to CDMS according to the same criteria as described above. CSF levels of apoAI were determined by ELISA (ELISAPRO kit for Human apolipoprotein AI; 3710-1HP-2; Mabtech AB, Nacka Strand, Sweden) in 74 CIS patients recruited at the Cemcat (27 CIS patients who remained with CIS during the follow-up period and 47 patients who converted to CDMS (cohort 3; Table 2)). Fifty individuals with OND were also included as controls (mean age = 42.9 (18.3); 64.6% females; cohort 3, Additional file 3: Table S3).

**Table 2:** **Clinical information and CSF characteristics of validation cohorts of CIS patients**

	Cohort 3* - ELISA studies			Cohort 4** - proteomic studies		
**Characteristics**	**CIS → CIS**	**CIS → CDMS**	***P*** **values**	**CIS → CIS**	**CIS → CDMS**	***P*** **values**
n	27	47	-	25	24	-
Age (years)^a^	30.5 (7.4)	31.3 (6.6)	0.706	36.5 (12.4)	34.2 (9.3)	0.509
Female/male (% female)	22/5 (81.5)	30/17 (63.8)	0.123	16/9 (64.0)	17/7 (70.8)	0.419
Follow-up time (years)^a^	4.2 (2.8)	8.8 (3.6)	8.24 × 10^−7^	3.2 (1.3)	10.7 (16.5)	0.034
Clinical presentation						
Optic neuritis	16 (59.3)	15 (31.9)	0.146	8 (32.0)	3 (12.5)	0.333
Brainstem	5 (18.5)	12 (25.5)		4 (16.0)	6 (29.1)	
Spinal	3 (11.1)	16 (34.0)		7 (28.0)	10 (41.6)	
Others	3 (11.1)	4 (8.5)		6 (24.0)	4 (16.6)	
CSF cells^b^	0.0 (0.0-2.0)	0.0 (0.0-4.0)	0.250	2.0 (0.0-3.2)	3.0 (1.7-9.5)	0.037
Proteins (mg/dL)^b^	30.0 (25.7-36.2)	34.0 (27.0-40.0)	0.297	28.0 (25.0-36.0)	30.0 (25.0-40.5)	0.753

*Cohort 3 was used for determination of CSF levels of apolipoprotein AI. **Cohort 4 was used for quantification of CSF levels of semaphorin 7A and ala-beta-his-dipeptidase. ^a^Data are expressed as mean (standard deviation). ^b^Data are expressed as median (interquartile range). *P* values were obtained following comparisons between CIS → CIS and CIS → CDMS patients by means of the chi-square test (gender and clinical presentation) and Mann-Whitney U test (remaining variables). Proteins (mg/dL) refers to protein concentration in mg/dL. CDMS, clinically definite multiple sclerosis; CIS, clinically isolated syndromes, CSF, cerebrospinal fluid.

The abundance of sema7A and CNDP1 in CSF samples were determined by SRM using the same parameters as detailed above in 49 CIS patients recruited at the Hospital Ramón y Cajal (Madrid, Spain); 24 CIS patients who converted to CDMS and 25 patients who remained with CIS during the follow-up period (cohort 4; Table 2). Twenty-two patients with OND were used as controls (mean age = 41.7 (12.5); 50% females; cohort 4, Additional file 3: Table S3). A summary of clinical information and CSF characteristics of CIS patients included in the validation cohorts is shown in Table 2.

### Statistical methods

Differences in CSF and serum protein levels detected by ELISA between CIS → CIS and CIS → CDMS patients, and between CIS patients and controls with OND, were evaluated by means of a Mann-Whitney U test. SRM peak intensities were normalized based on the isotopically labeled peptide standards and transformed by the logarithm base 2 and protein-level quantification, and testing for differential abundance were performed using a linear mixed-effects model as implemented in software package SRMstats [7].

## Results

### Cerebrospinal fluid semaphorin 7A, ala-beta-his-dipeptidase, and apolipoprotein AI levels in CIS patients who convert to CDMS

**Figure 1:**
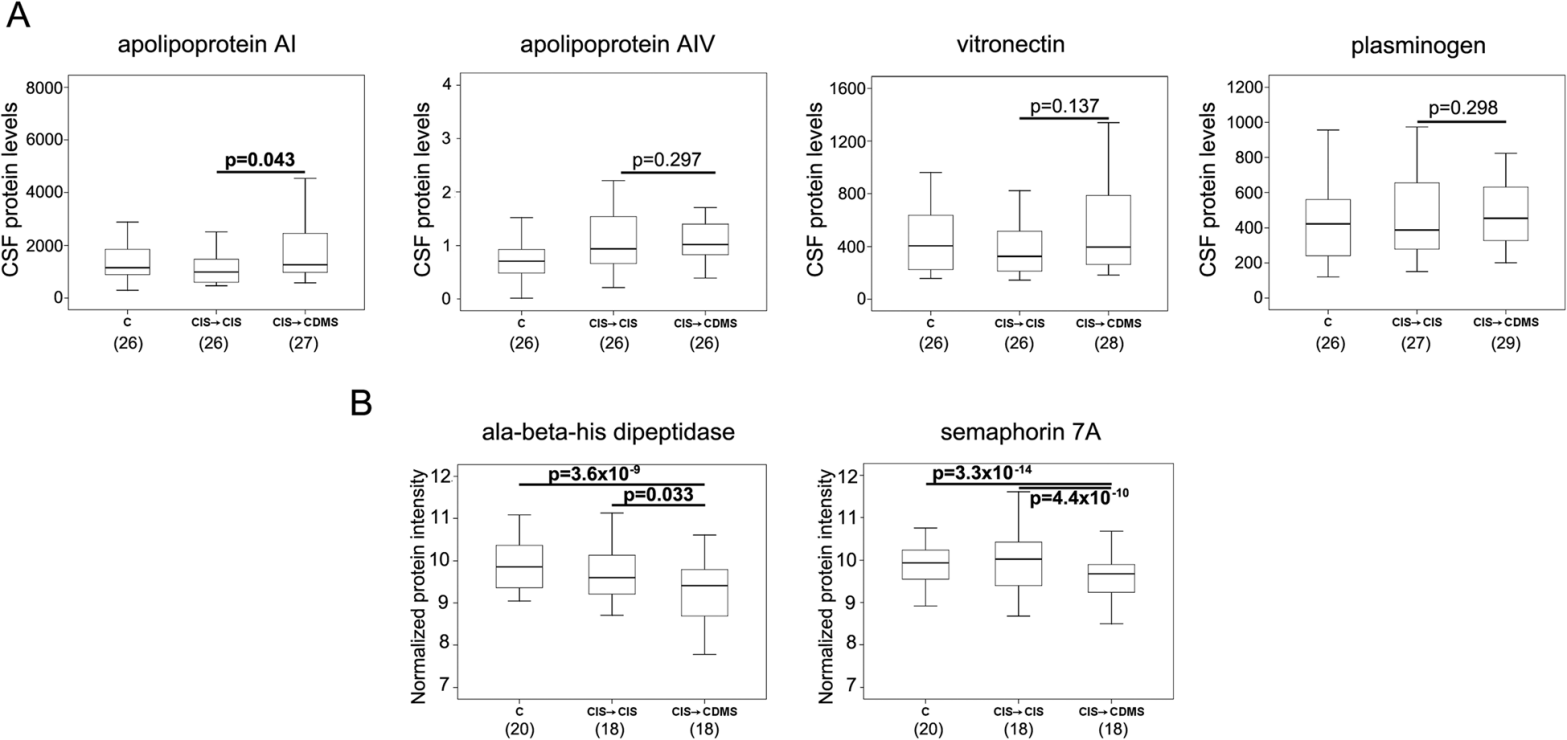
**CSF levels of apolipoprotein AI, apolipoprotein AIV, vitronectin, plasminogen, semaphorin 7A, and ala-beta-his-dipeptidase in a first and partially independent cohort of MS converters and non-converters. (A)** Boxplots showing CSF levels of apolipoprotein AI, apolipoprotein AIV, vitronectin, and plasminogen determined by commercially available ELISAs. **(B)** Boxplots representing CSF abundance of CNDP1 and semaphorin 7A determined by selected reaction monitoring. Statistically significant *P* values are shown in bold. *Indicates that significant differences were also observed between CIS → CDMS and controls (*P* = 3.3× 10^−14^ for semaphorin 7A and *P* = 3.6 × 10^−9^ for ala-beta-his dipeptidase). CIS → CIS: CIS patients who did not convert to clinically definite MS during the follow-up period. CIS → CDMS: CIS patients who converted to clinically definite MS. C: controls with other neurological disorders. Numbers in parentheses indicate individuals available for analysis. CDMS, clinically definite multiple sclerosis; CDNP1, ala-beta-his-dipeptidase; CIS, clinically isolated syndromes, CSF, cerebrospinal fluid.

Comparisons of CSF levels for candidate proteins in a partially independent cohort of CIS patients who converted to CDMS and CIS patients who remained with CIS revealed statistically significant differences for apoAI, sema7A, and CNDP1 between both groups of CIS patients. CSF apoAI levels determined by ELISA were significantly higher in CIS → CDMS patients than in CIS → CIS patients (*P* = 0.043; Figure 1A). On the other hand, CSF levels of sema7A and CNDP1 determined by SRM were significantly decreased in CIS → CDMS compared with CIS → CIS (*P* = 4.4 × 10^−10^ for sema7A and *P* = 0.033 for CNDP1; Figure 1B; Additional file 4: Table S4). Differences in CSF protein abundance were also observed following comparisons between CIS → CDMS patients and controls with OND (*P* = 3.3 × 10^−14^ for sema7A and *P* = 3.6 × 10^−9^ for CNDP1; Figure 1B; Additional file 4: Table S4). CSF levels of apoAIV, vitronectin, and plasminogen determined by ELISA were similar between CIS → CIS and CIS → CDMS patients (Figure 1A).

**Figure 2:**
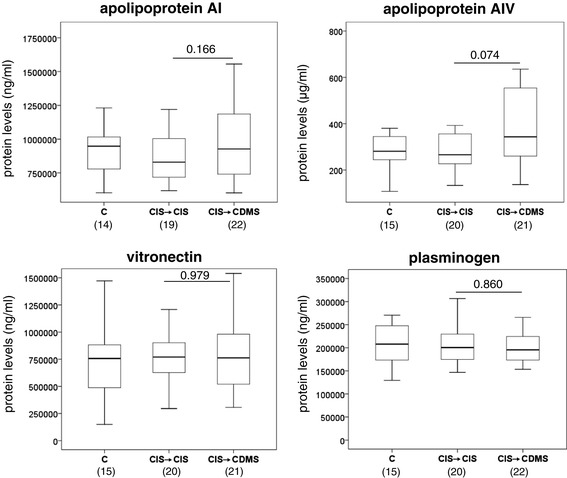
**Serum levels of apolipoprotein AI, apolipoprotein AIV, vitronectin, and plasminogen.** Boxplots showing serum protein levels determined by ELISA in CIS patients who converted to CDMS and CIS who remained with CIS. Outliers are represented with asterisks. Statistically significant p-values are shown in bold. CIS → CIS: CIS patients who did not convert to clinically definite MS during the follow-up period. CIS → CDMS: CIS patients who converted to clinically definite MS. C: controls with other neurological disorders. Numbers in parentheses indicate individuals available for analysis. CDMS, clinically definite multiple sclerosis; CIS, clinically isolated syndromes, CSF, cerebrospinal fluid.

Levels of apoAI, apoAIV, vitronectin, and plasminogen were also determined by ELISA in serum samples from the same cohort of CIS patients. However, no statistically significant differences were observed for any of the proteins between CIS patients who converted to CDMS and CIS patients who remained with CIS (Figure 2).

### Validation of semaphorin 7A and ala-beta-his-dipeptidase as biomarkers associated with conversion to CDMS in CIS patients

**Figure 3:**
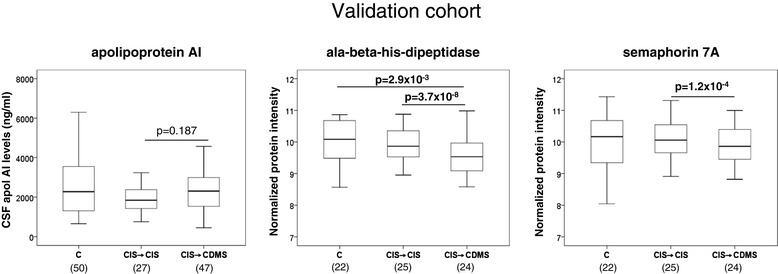
**Validation of apolipoprotein AI, semaphorin 7A, and ala-beta-his-dipeptidase as biomarkers associated with conversion to CDMS in a second and totally independent cohort of CIS patients.** Boxplots depicting CSF levels of apolipoprotein AI determined by ELISA, and CSF abundance of CNDP1 and semaphorin 7A determined by selected reaction monitoring in MS converters and non-converters to MS. Statistically significant *P* values are shown in bold. *Indicates that significant differences were also observed between CIS → CDMS and controls (*P* = 2.9 × 10^−3^). CIS → CIS: CIS patients who did not convert to clinically definite MS during the follow-up period. CIS → CDMS: CIS patients who converted to clinically definite MS. C: controls with other neurological disorders. Numbers in parentheses indicate individuals available for analysis. CDMS, clinically definite multiple sclerosis; CDNP1, ala-beta-his-dipeptidase; CIS, clinically isolated syndromes, CSF, cerebrospinal fluid.

In order to replicate CSF findings with apoAI, sema7A, and CNDP1, protein levels were also measured in a totally independent cohort of CIS patients who converted to CDMS and CIS patients who remained with CIS. As shown in Figure 3, CSF sema7A and CNDP1 levels were again found to be significantly decreased in CIS → CDMS patients compared with CIS → CIS patients (*P* = 1.2 × 10^−4^ for sema7A and *P* = 3.7 × 10^−8^ for CNDP1; Figure 3; Additional file 5: Table S5), thus confirming their association with conversion to CDMS. Differences in CSF CNDP levels were also observed following comparisons between CIS → CDMS patients and controls with OND (*P* = 2.9× 10^−3^; Figure 3; Additional file 5: Table S5). Regarding apoAI, although CSF mean protein levels were higher in CIS → CDMS patients than in CIS → CIS patients, differences did not reach statistical significance (*P* = 0.187; Figure 3). Analysis of CSF apoAI protein levels adjusted by follow-up time, which was significantly longer in the CIS → CDMS group compared with the CIS → CIS group (Table 2), resulted in a similar non-significant *P* value.

### Cerebrospinal fluid levels of semaphorin 7A are negatively associated with the number of T2 lesions at baseline and during the follow-up period

**Figure 4:**
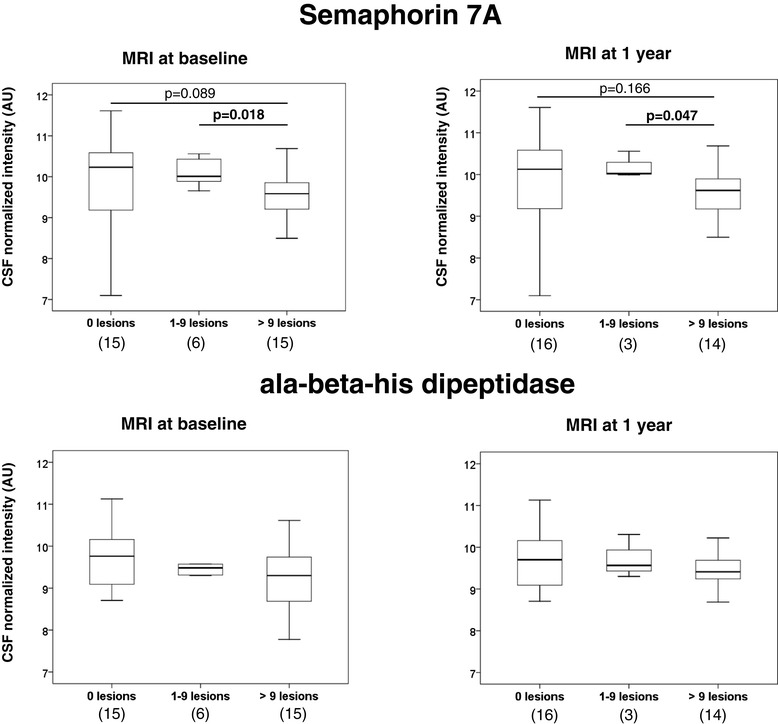
**CSF semaphorin 7A levels are negatively associated with MRI T2 lesion burden.** The number of T2 lesions at baseline and after one year of follow-up was grouped into three different categories: 0 lesions, between 1 and 9 lesions, or more than 9 lesions. Boxplots show the distribution of CSF levels of semaphorin 7A and ala-beta-his-dipeptidase among these categories. *P* values were obtained by means of a Mann-Whitney U test. Numbers in parentheses indicate individuals available for analysis. CSF, cerebrospinal fluid; MRI, magnetic resonance imaging.

As a last step, we aimed to correlate sema7A and CNDP1 findings with MRI abnormalities at baseline and during the follow-up period in the first cohort of CIS patients (Table 1). As shown in Figure 4, CSF sema7A levels negatively correlated with the number of T2 lesions observed in brain MRI scans performed at baseline and after one year of follow-up, and CSF protein levels decreased with an increasing number of T2 lesions. No significant differences were observed between CSF CNDP1 levels and the number of T2 lesions (Figure 4). Similarly, no significant correlations were found between CSF sema7A or CNDP1 levels and the number of gadolinium-enhancing lesions at baseline, or with number of gadolinium-enhancing lesions, number of new T2 lesions, and the Expanded Disability Status Scale score at one-year follow-up (data not shown).

Finally, correlations between CSF levels of sema7A and CNDP1 and CSF characteristics such as IgG index, CSF cells, or protein concentration were not statistically significant (data not shown).

## Discussion

In a previous proteomic study conducted by our group in pooled CSF samples, levels of apoAI, apoAIV, vitronectin, plasminogen, sema7A, and CNDP1 were found to be significantly different between CIS patients who converted to CDMS and patients who remained with CIS [5]. ApoAI was upregulated in CIS patients who converted to CDMS in all six CSF pools; apoAIV, vitronectin, and plasminogen were upregulated in four out of six pools. In contrast, sema7A and CNDP1 were downregulated in four out of six pools [5]. In the present study, we aimed to validate these proteins as biomarkers associated with the conversion to CDMS in individual CSF samples from CIS patients. Validation was performed in two stages, with a first replication study in CIS cohorts that had a partial overlap with the original pooled CIS cohort used for biomarker discovery (36% for cohort 1, 28% for cohort 2), and a second replication study conducted in CIS cohorts that were totally independent from the pooled CIS cohort. For all CIS patients, clinical and radiological criteria used to classify CIS patients into MS converters and non-converters were similar to the previous proteomic study [5]. Given the lack of commercially available ELISA assays for sema7A and CNDP1, CSF levels of these proteins were measured by SRM, a well-accepted sensitive, reproducible, and specific mass spectrometric technique for protein quantitation [8].

In the first replication step, three out of six candidates, apoAI, sema7A, and CNDP1, showed statistically significant differences between CIS patients who converted to CDMS and CIS patients who remained with CIS. In the second validation step, differences between MS converters and non-converters only remained significant for sema7A and CNDP1.

One of the most striking differences between MS converters and non-converters were observed for sema7A. Sema7A belongs to a family of membrane-bound and soluble proteins with roles in axonal guidance and immunomodulatory effects [9]. The decreased CSF levels of sema7A observed in MS converters may be related to its role as negative regulator of T-cell activation [10]. In this context, it has been shown that in sema7A-deficient mice, experimental autoimmune encephalomyelitis (EAE) disease course is more severe, EAE pathology is exacerbated, and T cells show increased proliferative responses to myelin oligodendrocyte glycoprotein antigen [10]. Interestingly, in our study CSF sema7A levels negatively correlated with the degree of lesion burden observed at baseline and during the follow-up period, finding that to be in line with the Czopik *et al*. study [10] where sema7A knockout mice showed increased inflammation and demyelination in the central nervous system compared with wild-type mice.

CNDP1 is a dipeptidase mainly expressed in the liver and brain that belongs to the M20 metalloprotease family [11]. CNDP1 hydrolyzes carnosine, which is known to have neuroprotective effects due to its capacity to decrease oxidative stress and inflammation [12-14]. Lower CSF levels of CNDP1 have been reported in MS patients compared with individuals with other neurological diseases [15], and a decrease in enzyme activity has been observed in serum samples from MS patients compared with healthy donors [16]. The lower CSF CNDP1 levels in CIS patients who later convert to CDMS may be associated with an increase in the levels of carnosine, which can be indirectly interpreted as an attempt to protect the brain from oxidative stress associated with higher inflammation in MS converters. However, the role of CSF CNDP1 in these patients needs further investigation, especially considering the lack of negative association observed in our study between inflammatory MRI abnormalities such as the number of gadolinium-enhancing lesions or T2 lesions and CSF CNDP1 levels.

Finally, the remaining proteins, (apoAI, apoAIV, vitronectin, and plasminogen) which were also proposed as candidate CSF biomarkers in the initial pooled proteomic study [5] were not validated in individual CSF samples using a different technique. Only apoAI, a specific inhibitor of proinflammatory cytokines secreted by activated T-cells [17], was found to be significantly increased in CSF from MS converters, but failed later validation in a totally independent cohort of CIS patients. Protein determinations in serum samples resulted in similar negative results. These proteins should therefore be considered as false positives found in the initial discovery proteomic approach [5].

Results from the present study validate two proteins, sema7A and CNDP1, as biomarkers associated with conversion to CDMS in CIS patients. These findings warrant further studies to investigate a potential association between decreased CSF levels of sema7A and CNDP1 and conversion to MS in CIS patients.

## Additional files

Additional file 1: Table S1 Demographic and clinical characteristics of patients with other neurological disorders. Additional file 2
**SRM acquisition method parameters.**
Additional file 3: Table S3 Demographic and clinical characteristics of patients with other neurological disorders. Additional file 4
**Intensities measured by SRM for all peptide ions measured in cohort 2 of patients.** These raw data were used to determine the fold changes and p-values referred to in the main text). Additional file 5
**Intensities measured by SRM for all peptide ions measured in cohort 4 of patients.** These raw data were used to determine the fold changes and p-values referred to in the main text).

## Abbreviations

ApoAI: apolipoprotein AI
ApoAIV: apolipoprotein AIV
CDMS: clinically definite MS
Cemcat: Centre d’Esclerosi Múltiple de Catalunya
CHI3L1: chitinase 3-like 1
CIS: clinically isolated syndrome
CNDP1: ala-beta-his-dipeptidase
CSF: cerebrospinal fluid
IgG: immunoglobulin G
MRI: magnetic resonance imaging
MS: multiple sclerosis
Sema7A: semaphorin 7A
SRM: selected reaction monitoring
OND: other neurological disorders
