# Supplementary material for: Validation of semaphorin 7A and ala-β-his-dipeptidase as biomarkers associated with the conversion from clinically isolated syndrome to multiple sclerosis
Source: J Neuroinflammation. 2014 Nov 13;11:181. doi: 10.1186/s12974-014-0181-8 (PMC4236472; doi:10.1186/s12974-014-0181-8)
Supplement: Additional file 1: Table S1 — Demographic and clinical characteristics of patients with other neurological disorders. [file 12974_2014_181_MOESM1_ESM.doc]

**Supplementary Table 1.** Demographic and clinical characteristics of patients with other neurological disorders

| Patients | Gender | Cohort 1*  Age | | Diagnosis | Patients | |  | Gender | Cohort 2*  Age | Diagnosis | |
| --- | --- | --- | --- | --- | --- | --- | --- | --- | --- | --- | --- |
| **1** | male | | 27.3 | myopathy | **1** | |  | male | 27.3 | myopathy | |
| **2** | female | | 50.9 | ↓ visual acuity | **2** | |  | female | 50.9 | ↓ visual acuity | |
| **3** | female | | 26.6 | pseudotumor cerebri | **3** | |  | female | 26.6 | pseudotumor cerebri | |
| **4** | male | | 47.5 | papillitis | **4** | |  | male | 47.5 | papillitis | |
| **5** | female | | 38.2 | pseudotumor cerebri | **5** | |  | female | 38.2 | pseudotumor cerebri | |
| **6** | male | | 55.1 | pseudotumor cerebri | **6** | |  | male | 55.1 | pseudotumor cerebri | |
| **7** | female | | 42.3 | hydrocephalus | **7** | |  | female | 42.3 | hydrocephalus | |
| **8** | female | | 23.7 | syncope | **8** | |  | female | 23.7 | syncope | |
| **9** | female | | 25.9 | papillitis | **9** | |  | female | 25.9 | papillitis | |
| **10** | female | | 30.7 | syncope | **10** | |  | female | 30.7 | syncope | |
| **11** | male | | 35.1 | papillitis | **11** | |  | male | 35.1 | papillitis | |
| **12** | male | | 64.6 | leukoencephalopathy | **12** | |  | male | 64.6 | leukoencephalopathy | |
| **13** | male | | 41.3 | headache | **13** | |  | male | 41.3 | headache | |
| **14** | male | | NA | vertigo | **14** | |  | male | *NA* | vertigo | |
| 15 | female | | 47.4 | myopathy | 15 | |  | male | 21.3 | headache | |
| 16 | female | | 19.7 | headache | 16 | |  | male | 76.1 | stroke | |
| 17 | male | | 48.8 | Guillain-Barré syndrome | 17 | |  | male | 47.6 | stroke | |
| 18 | female | | 21.6 | neck pain | 18 | |  | female | 48.4 | radiculopathy | |
| 19 | *NA* | | *NA* | papillitis | 19 | |  | female | 25.4 | pseudotumor cerebri | |
| 20 | *NA* | | *NA* | stroke | 20 | |  | male | 59.0 | optic neuropathy | |
| 21 | female | | 25.6 | diplopia |  |  |  | |  | |  |
| 22 | male | | 62.9 | headache |  |  |  | |  | |  |
| 23 | *NA* | | *NA* | stroke |  |  |  | |  | |  |
| 24 | *NA* | | *NA* | stroke |  |  |  | |  | |  |
| 25 | *NA* | | *NA* | headache |  |  |  | |  | |  |
| 26 | female | | 20.2 | ↓ visual acuity |  |  |  | |  | |  |

*Cohort 1 was used for determination of CSF and serum levels of apoAI, apoAIV, vitronectin and plasminogen by ELISA. *Cohort 2 was used for quantification of CSF levels of sema7A and CNDP1 by selected reaction monitoring. Numbers in bold refers to individuals that were present in both cohorts of controls with other neurological disorders. NA: information was not available.
