# Supplementary material for: Validation of semaphorin 7A and ala-β-his-dipeptidase as biomarkers associated with the conversion from clinically isolated syndrome to multiple sclerosis
Source: J Neuroinflammation. 2014 Nov 13;11:181. doi: 10.1186/s12974-014-0181-8 (PMC4236472; doi:10.1186/s12974-014-0181-8)
Supplement: Additional file 3: Table S3 — Demographic and clinical characteristics of patients with other neurological disorders. [file 12974_2014_181_MOESM3_ESM.doc]

**Supplementary Table 3.** Demographic and clinical characteristics of patients with other neurological disorders

| Patients | Gender | Cohort 3*  Age | | Diagnosis | Patients | Gender | Cohort 4*  Age | | Diagnosis |
| --- | --- | --- | --- | --- | --- | --- | --- | --- | --- |
| 1 | male | 59 | optic neuropathy | | 1 | female | 31 | pseudotumor cerebri | |
| 2 | male | 32 | paraparesis | | 2 | female | 48 | headache | |
| 3 | female | 25 | parasthesia | | 3 | female | 45 | toxic encephalopathy | |
| 4 | female | 20 | polyneuropathy | | 4 | male | 57 | ictus | |
| 5 | female | 38 | hemiparesis | | 5 | male | 36 | Headache | |
| 6 | female | 42 | optic neuropathy | | 6 | female | 37 | diabetic polyneuropathy | |
| 7 | male | 44 | cerebellar ataxia | | 7 | female | 44 | PT muscle contracture | |
| 8 | male | 23 | facial paralysis | | 8 | female | 57 | confusional syndrome | |
| 9 | female | 75 | paraparesis | | 9 | male | 23 | headache | |
| 10 | female | 51 | migraine | | 10 | male | 17 | epilepsy | |
| 11 | female | 45 | sensory disturbances | | 11 | female | 32 | stroke | |
| 12 | male | 79 | polineuropathy | | 12 | female | 55 | epilepsy | |
| 13 | male | 28 | optic neuropathy | | 13 | female | 24 | headache | |
| 14 | female | 27 | paresthesia | | 14 | male | 39 | NP hydrocephalus | |
| 15 | female | 83 | multifocal motor neuropathy | | 15 | male | 24 | headache | |
| 16 | *NA* | *NA* | headache | | 16 | male | 51 | headache | |
| 17 | female | 26 | diplopia | | 17 | male | 52 | diabetic polyneuropathy | |
| 18 | female | 20 | meningitis | | 18 | male | 52 | ALS | |
| 19 | *NA* | *NA* | herpes simples encephalitis | | 19 | female | 45 | pseudotumor cerebri | |
| 20 | male | 63 | recurrent optic neuritis | | 20 | female | 37 | headache | |
| 21 | female | 68 | motor neuron disease | | 21 | male | 52 | papilledema | |
| 22 | female | 24 | vasculitis | | 22 | male | 59 | hydrocephalus | |
| 23 | female | 27 | motor-sensory syndrome | |  |  |  |  | |
| 24 | female | 20 | hemiparesis | |  |  |  |  | |
| 25 | female | 33 | fibromyalgia | |  |  |  |  | |
| 26 | female | 41 | polyneuropathy | |  |  |  |  | |
| 27 | female | 73 | antiphospholipid syndrome | |  |  |  |  | |
| 28 | female | 61 | ↓ visual acuity | |  |  |  |  | |
| 29 | female | 31 | headache and diplopia | |  |  |  |  | |
| 30 | female | 45 | optic neuropathy | |  |  |  |  | |
| 31 | female | 22 | Miller-Fisher syndrome | |  |  |  |  | |
| 32 | female | 24 | peripheral facial palsy | |  |  |  |  | |
| 33 | female | 32 | peripheral facial palsy | |  |  |  |  | |
| 34 | female | 60 | ↓ visual acuity | |  |  |  |  | |
| 35 | male | 39 | headache | |  |  |  |  | |
| 36 | male | 41 | stroke | |  |  |  |  | |
| 37 | male | 70 | Parkinson's disease | |  |  |  |  | |
| 38 | male | 61 | confusional syndrome | |  |  |  |  | |
| 39 | female | 34 | diplopia | |  |  |  |  | |
| 40 | male | 37 | stroke | |  |  |  |  | |
| 41 | male | 24 | encephalopathy | |  |  |  |  | |
| 42 | female | 65 | optic neuropathy | |  |  |  |  | |
| 43 | female | 25 | polyneuropathy | |  |  |  |  | |
| 44 | male | 65 | multifocal necrotizing leucoencephalopathy | |  |  |  |  | |
| 45 | female | 33 | sensory disturbances | |  |  |  |  | |
| 46 | female | 67 | spastic paraparesis | |  |  |  |  | |
| 47 | male |  | vertigo | |  |  |  |  | |
| 48 | male | 32 | diplopia | |  |  |  |  | |
| 49 | female | 46 | sensory hemisyndrome | |  |  |  |  | |
| 50 | male | 39 | peripheral facial palsy | |  |  |  |  | |

*Cohort 3 was used for determination of CSF levels of apoAI by ELISA. *Cohort 4 was used for quantification of CSF levels of sema7A and CNDP1 by selected monitoring reaction. NA: information was not available. ALS: amyotrophic lateral sclerosis. NP hydrocephalus: normal pressure hydrocephalus. PT muscle contracture: post-traumatic muscle contracture.
